# Exercise performance is not improved in mice with skeletal muscle deletion of natriuretic peptide clearance receptor

**DOI:** 10.1371/journal.pone.0293636

**Published:** 2023-11-02

**Authors:** Brigitte Jia, Alexander Hasse, Fubiao Shi, Sheila Collins

**Affiliations:** 1 Division of Cardiovascular Medicine, Department of Medicine, Vanderbilt University Medical Center, Nashville, TN, United States of America; 2 Sanford Burnham Prebys Medical Discovery Institute, Orlando, FL, United States of America; 3 Department of Molecular Physiology and Biophysics, Vanderbilt University, Nashville TN, United States of America; Kindai University Faculty of Medicine, JAPAN

## Abstract

Natriuretic peptides (NP), including atrial, brain, and C-type natriuretic peptides (ANP, BNP, and CNP), play essential roles in regulating blood pressure, cardiovascular homeostasis, and systemic metabolism. One of the major metabolic effects of NP is manifested by their capacity to stimulate lipolysis and the thermogenesis gene program in adipocytes, however, in skeletal muscle their effects on metabolism and muscle function are not as well understood. There are three NP receptors (NPR): NPRA, NPRB, and NPRC, and all three NPR genes are expressed in skeletal muscle and C2C12 myocytes. In C2C12 myocytes treatment with either ANP, BNP, or CNP evokes the cGMP signaling pathway. Since NPRC functions as a clearance receptor and the amount of NPRC in a cell type determines the signaling strength of NPs, we generated a genetic model with *Nprc* gene deletion in skeletal muscle and tested whether enhancing NP signaling by preventing its clearance in skeletal muscle would improve exercise performance in mice. Under sedentary conditions, *Nprc* skeletal muscle knockout (MKO) mice showed comparable exercise performance to their floxed littermates in terms of maximal running velocity and total endurance running time. Eight weeks of voluntary running-wheel training in a young cohort significantly increased exercise performance, but no significant differences were observed in MKO compared with floxed control mice. Furthermore, 6-weeks of treadmill training in a relatively aged cohort also increased exercise performance compared with their baseline values, but again there were no differences between genotypes. In summary, our study suggests that NP signaling is potentially important in skeletal myocytes but its function in skeletal muscle *in vivo* needs to be further studied in additional physiological conditions or with new genetic mouse models.

## Introduction

The cardiac natriuretic peptides (NP) atrial natriuretic peptide (ANP) and B-type natriuretic peptide (BNP) are hormones that are produced in the heart, while C-type natriuretic peptide (CNP) is expressed more broadly, including in the central nervous system and vasculature. They play essential roles in blood pressure control, cardiovascular homeostasis, and systemic metabolic regulation. There are three types of NP receptors (NPR), including the guanylyl cyclase-coupled receptors NPRA and NPRB, and the clearance receptor NPRC. ANP and BNP bind to the NPRA receptor, while CNP binds to the NPRB receptor. All three peptides bind with relatively equal affinity to NPRC, a transmembrane receptor that does not have an intracellular guanylyl cyclase domain [1]. Binding of NP with NPRA and NPRB receptors activates their intracellular guanylyl cyclase activity to stimulate cyclic guanosine monophosphate (cGMP) production and increase cGMP-dependent protein kinase (PKG) phosphorylation of downstream targets. In contrast, binding with the NPRC receptor internalizes the peptide for degradation and thus effectively clears the peptide from circulation [2]. Recently we reported that another means by which NPRC can blunt cGMP production is by the formation of non-functional heterodimers between NPRA-NPRC and NPRB-NPRC [3].

Exercise, a well-known modulator of bodyweight and systemic metabolism, can reduce the risk of developing obesity-related conditions such as Type 2 diabetes and cardiovascular disease [4]. Rising obesity rates and accompanying co-morbidities in the population necessitate the ongoing search for ways to improve the beneficial metabolic effects of exercise on the human body. In healthy human subjects, prolonged endurance exercise has been shown to increase plasma NP levels [5]. Previous reports have shown that NPs are inversely related to visceral fat, body mass index, and circulating insulin, and widely thought to influence obesity via their lipolytic effects in adipose tissue, fatty acid oxidation in skeletal muscle, and adipose tissue [6]. In adipose tissues, NPs can increase adipose tissue ‘browning’ and mitochondrial biogenesis through PKG-mediated activation of peroxisome proliferator activated receptor gamma coactivator 1-α (PGC1α) and uncoupling protein 1 (UCP1) [7, 8].

The NP clearance receptor NPRC plays essential roles in dictating NP signaling and its metabolic effect on target tissues. We previously reported that deletion of *Nprc* from adipose tissue in mice led to protection from diet-induced obesity, increased energy expenditure and glucose uptake into brown fat, and overall improved NP signaling in the adipose tissue [7, 8]. These improvements occurred without any change in blood pressure or circulating NPs. Therefore, we were interested to learn whether the metabolic benefit of exercise for obesity intervention could be mediated by similarly increasing NP signaling in skeletal muscle by removing NPRC. In addition, NP treatment has been reported to increase mitochondrial metabolism, fat oxidation, and maximal respiration in human myotubes [9]. *NPRA* (*NPR1*) gene expression is positively correlated to mRNA levels of *PGC1α* and several oxidative phosphorylation (OXPHOS) genes in human skeletal muscle, and the expression of *NPRA*, *PGC1α*, and OXPHOS genes is coordinately upregulated in response to aerobic exercise training in human skeletal muscle [9]. These studies suggest a role for NP signaling in improving mitochondrial function and oxidative metabolism in muscle [9]. Further studies of the metabolic function of NPs in skeletal muscle tissue could shed more light on their ability to combat obesity and glucose intolerance. In the work described here we sought to address the potential benefit of NPRC deficiency in skeletal muscle to determine whether it would improve the exercise performance.

## Materials and methods

### Reagents and antibodies

The membrane-permeable 8-Br-cGMP (8-bromoguanosine 3′,5′-cyclic monophosphate, B1381) and 8-Br-cAMP (8-bromoadenosine 3′,5′-cyclic monophosphate, B5386) were from Sigma. ANP (AS-20648), BNP (AS-24016), and CNP (AS-24244) were from AnaSpec. Antibodies used in this study included anti-p-Ser239 VASP (3114, Cell Signaling Technology), anti-total VASP (3112, Cell Signaling Technology), anti-GAPDH (sc25788, Santa Cruz; 10494-I-AP, ProteinTech), anti-rabbit IgG conjugated with alkaline phosphatase (A3687, Sigma).

### Cell lines

C2C12 mouse myoblast cells (ATCC, CRL-1772) were cultured in DMEM (4.5g/L of L-glucose, no sodium pyruvate) with 10% FBS, 1% penicillin-streptomycin. For differentiation, C2C12 cells were allowed to grow to confluency and differentiated in DMEM with 2% donor equine serum, 1μM insulin, 1% penicillin-streptomycin for 6 days.

### Mice

Mice with a floxed *Nprc* allele were generated as previously described (7). *Myogenin*-Cre mice were a gift from Eric Olson of University of Texas Southwestern Medical Center and bred with *Nprc* floxed mice to generate skeletal muscle specific knockout (MKO) mice. Mice were kept under a 12-hour light/12-hour dark cycle at constant temperature (23°C) with ad libitum access to water and mouse chow diet. All animal studies were approved by the Institutional Animal Care and Use Committees of Sanford Burnham Prebys Medical Discovery Institute and Vanderbilt University Medical Center in accordance with the National Institutes of Health (NIH) Guide for the Care and Use of Laboratory Animals. Animals were not subjected to any painful procedures that required anesthesia and/or analgesia. Tissues were collected from animals following CO_2_ euthanasia.

### Exercise performance and training

#### Cohort 1: Sprint and endurance tests

Three- to 5-month-old Nprc MKO and control male mice were tested on an ECO-6M Treadmill (Columbus Instruments, Columbus, OH) with a 10 min warm-up at a speed of 10 m/min. For endurance test, the speed was then increased by 2 m/min every 5 min until reaching a final constant speed of 20 m/min. For sprint test, the speed was increased by 2 m/min every 2 min until the mice were exhausted. The point of exhaustion for both tests was reached when mice made continuous contact with the shock grid for 5 seconds.

#### Cohort 2: Running-wheel training

Six-weeks-old *Nprc* MKO and control male mice were individually housed in a cage with a running-wheel outfitted with an odometer for eight weeks. The daily running distance were recorded with the odometer. After 8-weeks training, mice were subjected to endurance and sprint tests as described in Cohort 1.

#### Cohort 3: Treadmill training

Ten-month-old *Nprc* MKO and control mice underwent a seven-day adaptation training procedure. For adaptation training, mice were placed on the treadmills for a total of 45 minutes, with an initial speed at 8m/min for the first 10 minutes and then with a constant speed at 10 m/min afterwards. Mice that failed to complete at least five days of the adaptation training were removed from the study. After adaptation training, endurance and sprint tests were performed to determine the baseline performance. For endurance test, treadmill speed was initially set at 8 m/min and then increased by 1 m/min every 10 minute until exhaustion. For sprint test, treadmill speed was set at 8 m/min for 10 minutes and then increased by 1 m/min every 4 minutes until exhaustion. After baseline tests, mice were trained at 60% of their average maximum speeds for 1 h/day and 5 d/week for 3 weeks. At 3 weeks of training, mice underwent a second sprint test and trained at 60% of adjusted average maximum speeds for 1 h/day and 5 d/week for another 3 weeks. After 6 weeks of training in total, mice underwent final endurance and sprint tests to determine the final performance.

### RT-qPCR

Total RNA was extracted from C2C12 cells or tissues using TRIzol reagent (Invitrogen). C2C12 samples were then purified by isopropanol precipitation, and tissue samples by RNeasy Mini (Qiagen) or Quick-RNA Miniprep (Zymo Research) kits. cDNA was prepared with the High-Capacity cDNA Reverse Transcription (RT) kit (Applied Biology). Quantitative PCR (qPCR) were conducted with PowerUp SYBR Green Master Mix (Life Technologies) on the QuantStudio 6 Flex System (Applied Biosystems) according to the manufacturer’s protocols. The sequences of primers are listed in **S1 Table**. RT-qPCR results were analyzed by ΔΔCt method, normalized to the reference gene, and expressed as mean ± SEM. *Ap3d1* and *36B4* were used as the reference genes for C2C12 cells and tissue samples, respectively.

### Western blot

Differentiated C2C12 cells were serum-starved for 48h in low-glucose Gibco DMEM and then stimulated with ANP, BNP, or CNP at 200nM for 30min, or subjected to a 30min treatment with 200nM 8-Br-cGMP as a positive control to induce downstream PKG activity and 200nM 8-Br-cAMP to act as a negative control. Cells were lysed and sonicated in lysis buffer (25 mM HEPES at pH 7.4, 150 mM NaCl, 5 mM EDTA, 5 mM EGTA, 5 mM glycerophosphate, 0.9% Triton X-100, 0.1% IGEPAL, 5 mM sodium pyrophosphate, 10% glycerol, 1x Complete protease inhibitor and 1x PhoSTOP phosphatase inhibitor cocktail). For Western blotting, 40 μg of protein was resolved by 10% SDS-polyacrylamide gel electrophoresis, transferred to nitrocellulose membranes (Bio-Rad), incubated overnight at 4°C with primary antibodies, and followed by inoculation with alkaline phosphatase–conjugated secondary antibody for 1 hour at room temperature. Blots were incubated with Amersham ECF substrate (Cytiva, RPN5785) and images were acquired by Bio-Rad digital ChemiDoc MP with IR (VUMC Molecular Cell Biology Resource).

### Statistical analysis

GraphPad Prism 7 was used for statistical analysis. All data were presented as means ± SEM. Two-way ANOVA were used to determine the differences as indicated. Statistical significance was defined as p-value < 0.05.

## Results

### NP receptor gene expression in C2C12 myocytes

**Fig 1:**
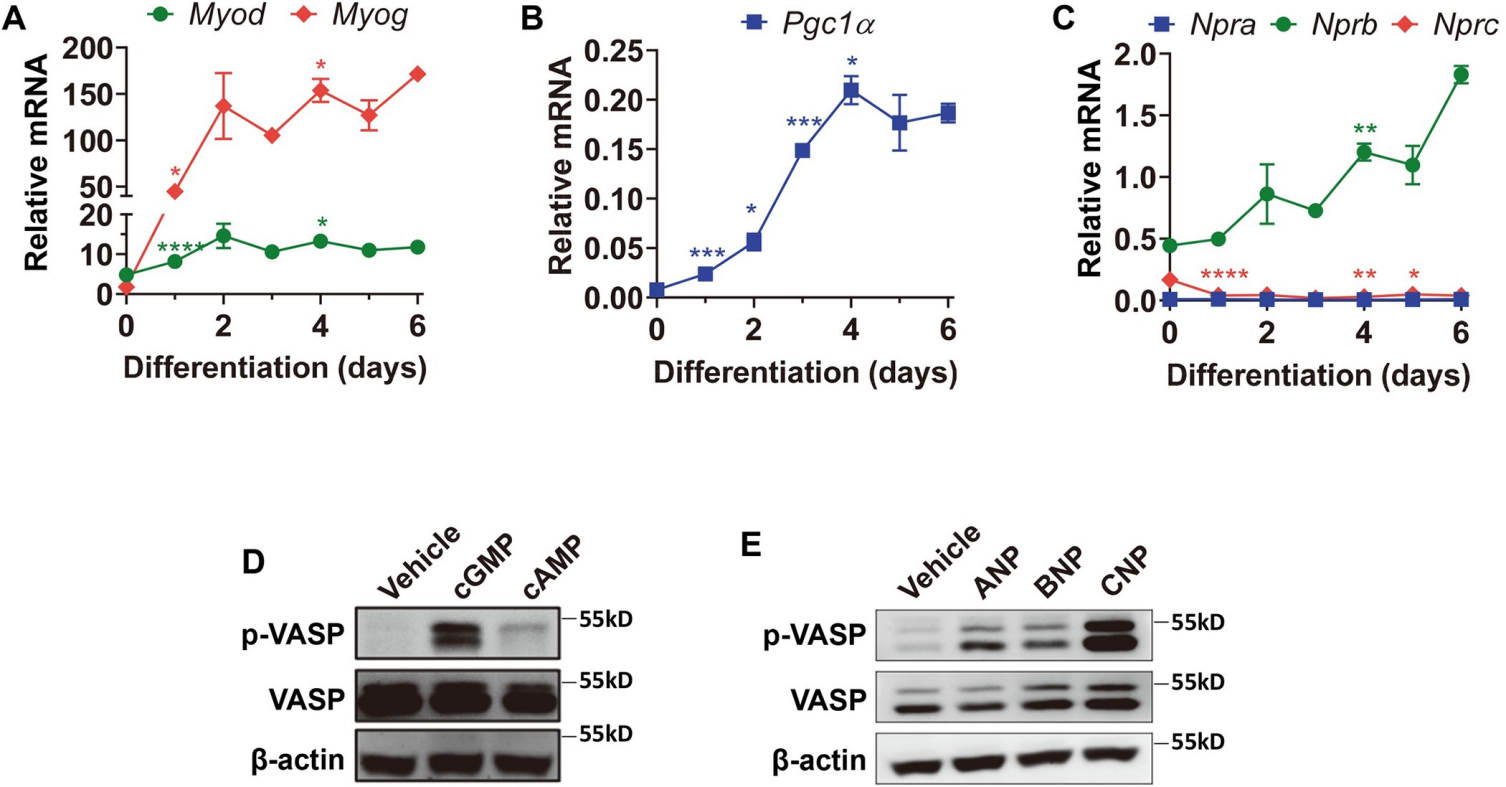
Expression of natriuretic peptide receptor genes in C2C12 myocytes and activation of PKG by NPs. The mRNA levels of (A) myocyte marker genes *Myod* (B), *Pgc1α* and NP receptor genes *Npra*, *Nprb* and *Nprc* in differentiating C2C12 myocytes. Data were normalized to internal control gene *36B4*. Unpaired two-tailed Student’s t-test, compared with previous day, *p<0.05, **p<0.01, ***p<0.001, ****p<0.0001. After differentiation, C2C12 myocytes were serum-starved overnight and treated (D) with membrane-permeable 8-Br-cGMP (200nM) or 8-Br-cAMP (200nM); (E) with ANP (200 nM), BNP (200 nM) or CNP (200 nM) for 30 minutes. Levels of phosphorylated VASP (p-VASP S239), total VASP (VASP), and β-actin were then measured by Western blot analysis.

To explore the role of natriuretic peptide signaling in regulating skeletal muscle function and metabolism, we began by first examining the expression of natriuretic peptide receptor genes in C2C12 cells, a commonly used myocyte cell line. The expression of myocyte genes (**Fig 1A**) and the transcriptional co-regulator PGC1α (**Fig 1B**) were increased during the course of differentiation of C2C12 cells. As shown in **Fig 1C**, the expression of NP receptor A (*Npra*, or *Npr1*) did not change, while the expression of NP receptor C (*Nprc*, or *Npr3*) tended to decrease. The expression of NP receptor B (*Nprb*, or *Npr2*) was more robustly increased, as also observed in a previous report [10].

### NPs stimulate PKG signaling in C2C12 myocytes

To further determine the effect of NPs on C2C12 myocytes, we next checked NP-stimulated cGMP signaling in C2C12 myocytes. Treatment with a membrane-permeable analog of cyclic guanosine monophosphate (cGMP), but not cyclic adenosine monophosphate (cAMP), increased the phosphorylation of Ser-239 (p-S^239^) in vasodilator-stimulated phosphoprotein (VASP) (**Fig 1D** and **S1 Fig**). The Ser-239 site is well established to be phosphorylated by protein kinase G (PKG), but not by PKA [11]. Similarly, treatment with either ANP, BNP, or CNP increased p-S^239^ in VASP. The effect of CNP on p-S^239^ in VASP is the most robust among the three NPs (**Fig 1E** and **S2 Fig**), which is consistent with the observation that NPRB expression is the highest among the three NPRs in fully differentiated C2C12 myocytes (**Fig 1C**). These data demonstrate that NPs can exert functional effects on C2C12 myocytes through the NPR-PKG signaling cascade.

### Expression of *Nprc* in skeletal muscle groups

**Fig 2:**
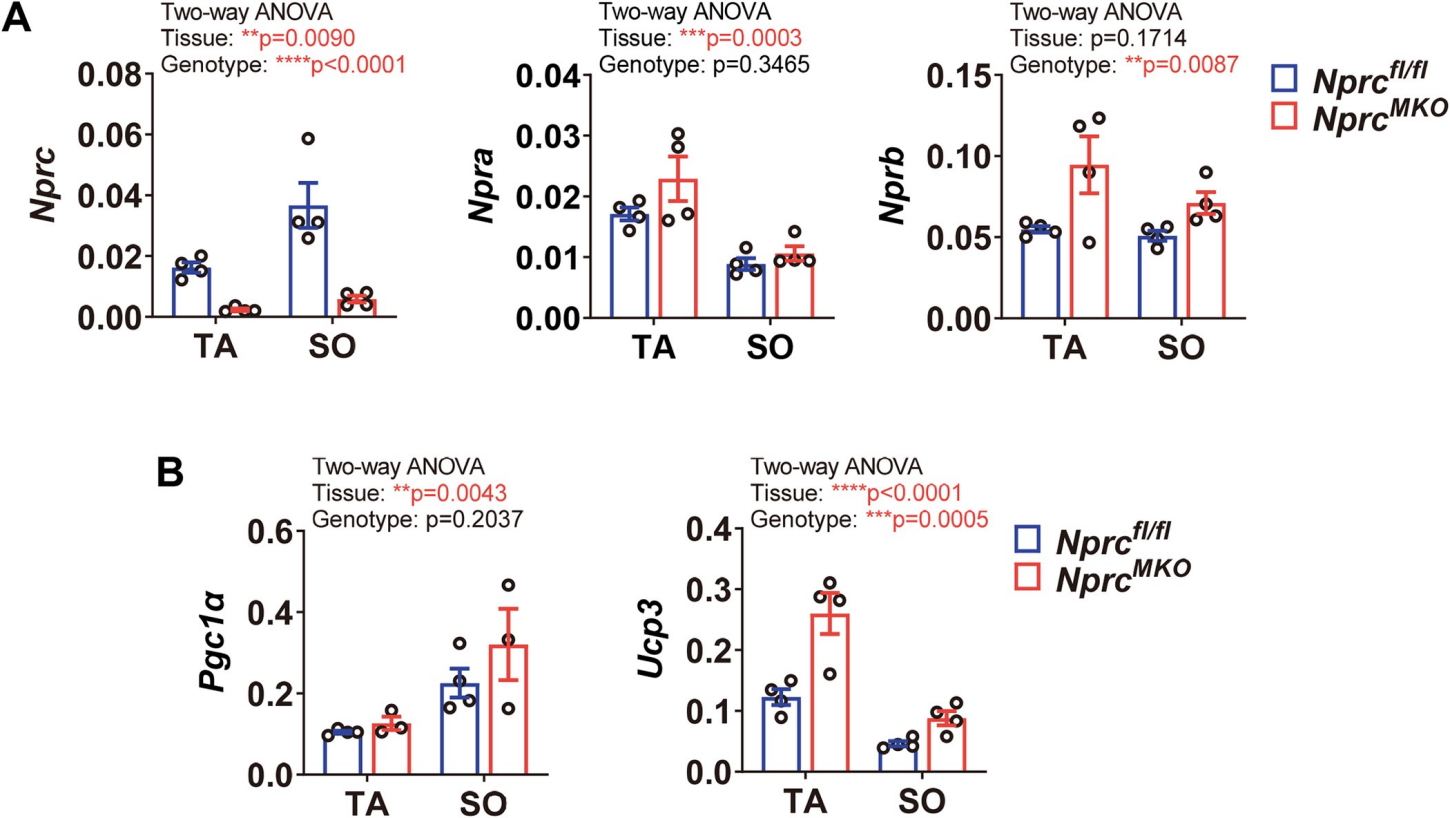
Gene expression profile in skeletal muscle of sedentary mice. (A) The mRNA levels of (A) NP receptor genes *Nprc*, *Npra* and *Nprb*; (B) *Pgc1α* and *Ucp3* in the tibialis anterior (TA) and soleus (SO) muscles of control and *Nprc* MKO mice. Two-way ANOVA, ** p < 0.01; *** p < 0.001; **** p < 0.0001; and p>0.05, no statistical significance.

As shown in our previous study [7], the expression of *Nprc* is relatively low in quadriceps femoris (QU), gastrocnemius (GA) and extensor digitorum longus (EDL) muscles, moderate in tibialis anterior (TA) muscle, and the highest in soleus muscle, while the expression of *Npra* is more consistent between these different types of muscles [7]. In this regard, our gene expression analysis in the current manuscript focused primarily on the TA and soleus muscles. The *Nprc* MKO mice grow normally and do not display any gross morphological changes. As expected, the expression of *Nprc* was essentially absent in the TA and soleus muscles of the MKO mice (**Fig 2A**). The expression of *Npra* did not change, but the expression of *Nprb* tended to increase in the skeletal muscle of *Nprc* MKO mice (**Fig 2A**). The expression of *Pgc1α*, a transcriptional co-regulator involved in mitochondrial biogenesis, was comparable between genotypes in both TA and soleus muscles (**Fig 2B**). Interestingly, the expression of *Uncoupling protein 3* (*Ucp3*), a gene known to be highly expressed in skeletal muscle [12], was significantly increased in both TA and soleus muscles of the *Nprc* MKO mice (**Fig 2B**). UCP3, along with its close homologue UCP2, are known to be increased in tissues in response to oxidative stress [13–15], and greater UCP3 expression can also result from increased mitochondrial fatty acid metabolism (reviewed in [16].

### Exercise performance of *Nprc* MKO after running-wheel training

**Fig 3:**
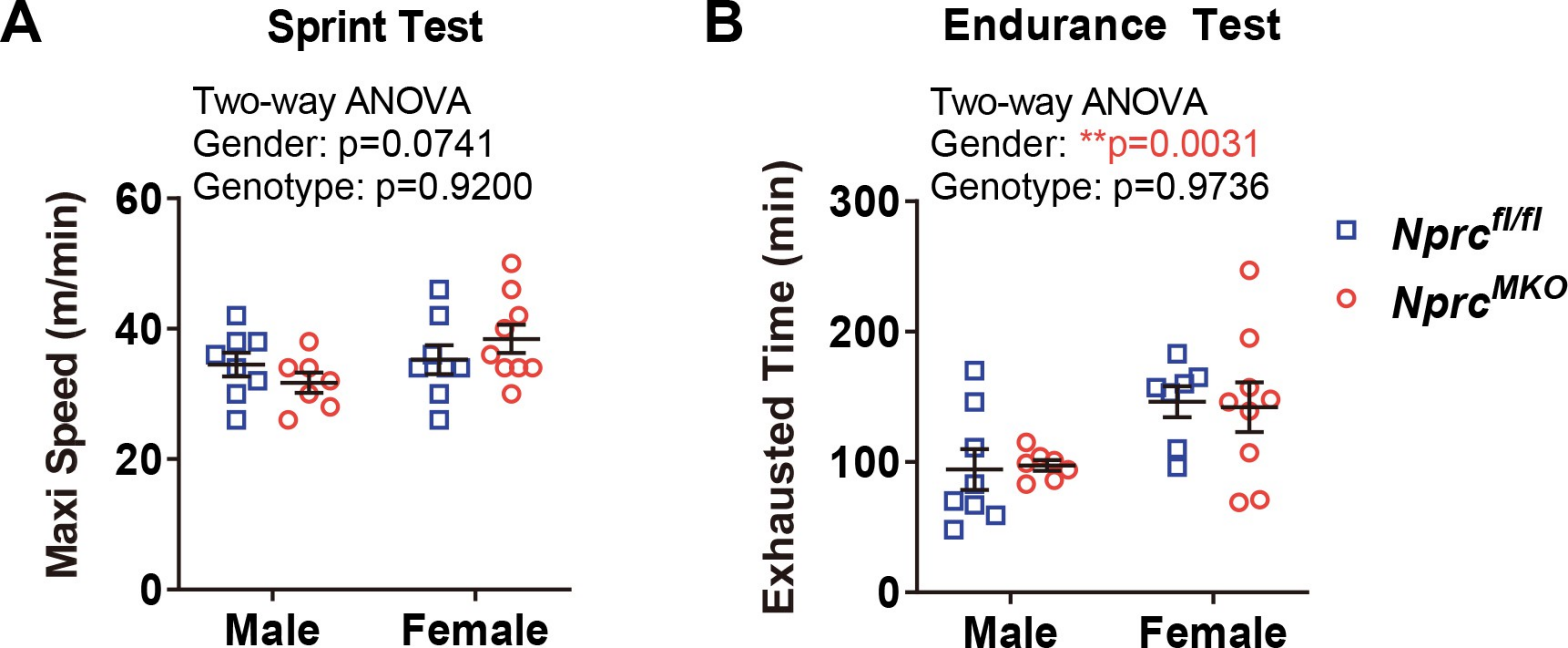
Exercise performance at sedentary status. A cohort of 3.5- to 5-month-old *Nprc* MKO and control mice were subjected to (A) sprint exercise test and (B) endurance exercise test to determine their maximal running speed and exhausted running time, respectively. Two-way ANOVA, ** p < 0.01; p>0.05, no statistical significance.

**Fig 4:**
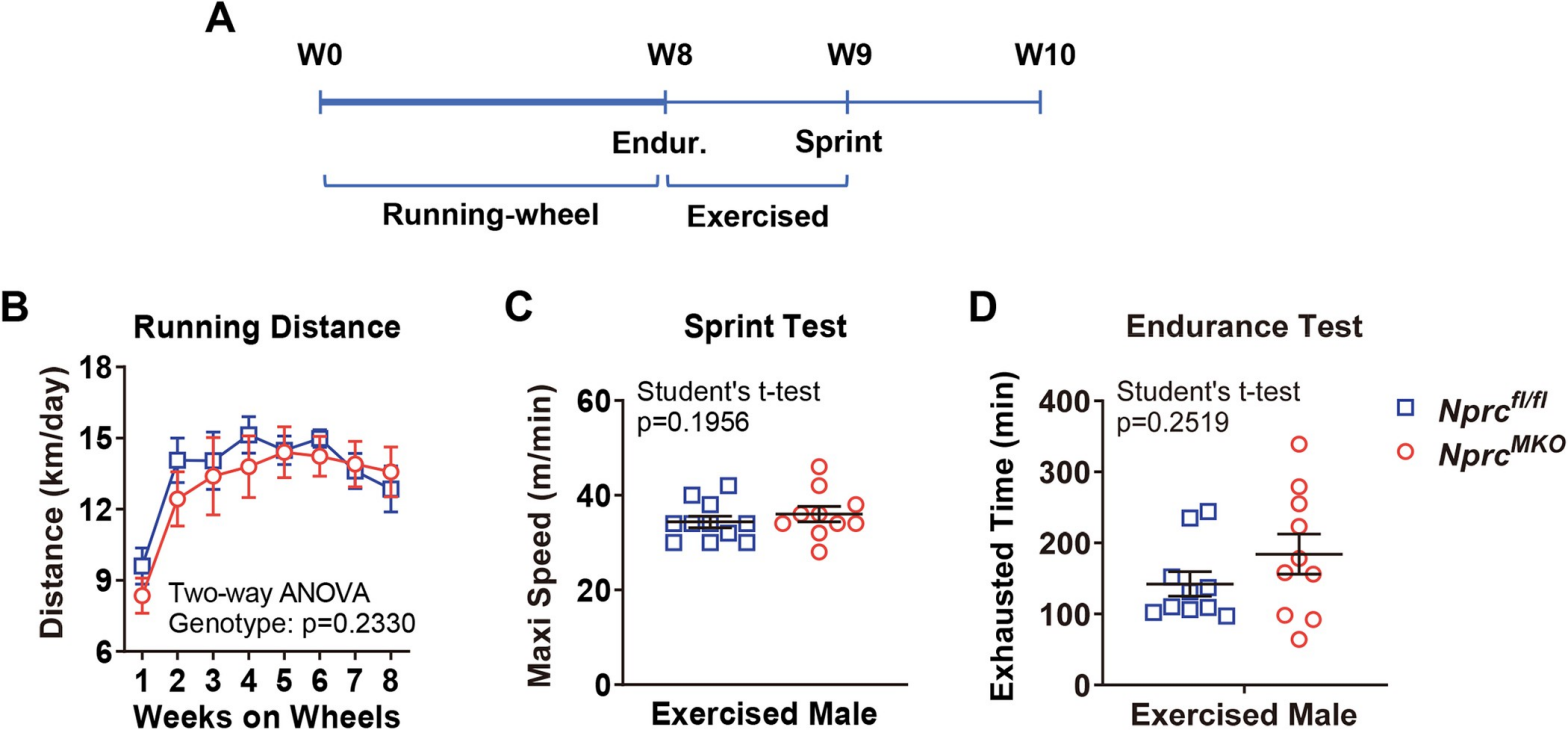
Exercise performance after 8 weeks of running-wheel training. (A) Timelines of running wheel training, endurance (Endur.) and sprint exercise tests. Six-week-old *Nprc* MKO and control male mice were trained in cages with a running-wheel outfitted with an odometer for eight weeks. (B) The daily running distances during running-wheel training. After 8 weeks of training, mice were subjected to (C) sprint exercise test and (D) endurance exercise test to determine their maximal running speed and exhausted running time, respectively. Two-way ANOVA as indicated, p>0.05, no statistical significance.

Levels of cardiac natriuretic peptides have been reported to increase in healthy individuals after exercise training [5, 17–19]. Since NPs are reported to increase fat oxidation in humans skeletal muscle [9, 20] as well as in mice [21], including being augmented in whole-body *Nprc-/-* mice [22], we next sought to examine whether exercise performance of *Nprc* MKO mice would be improved in the context of exercise training. We first checked the exercise performance of a sedentary cohort of 3- to 4-month-old male mice but did not observe any differences between genotypes regarding the maximal running speed and total endurance running time (**Fig 3A and 3B**). We next set up a second cohort of mice for exercise training on voluntary running-wheels for eight weeks (**Fig 4A**). Both *Nprc* MKO and control mice underwent similar amount of exercise training, as suggested by comparable running distance between two genotype group (**Fig 4B**). Their exercise capacity after training was evaluated as shown in the sprint (**Fig 4C**) and endurance exercise (**Fig 4D**) tests. However, both the maximal running speed and endurance running distance of *Nprc* MKO was not improved compared to that of the control mice.

### Exercise performance of *Nprc* MKO after treadmill training

**Fig 5:**
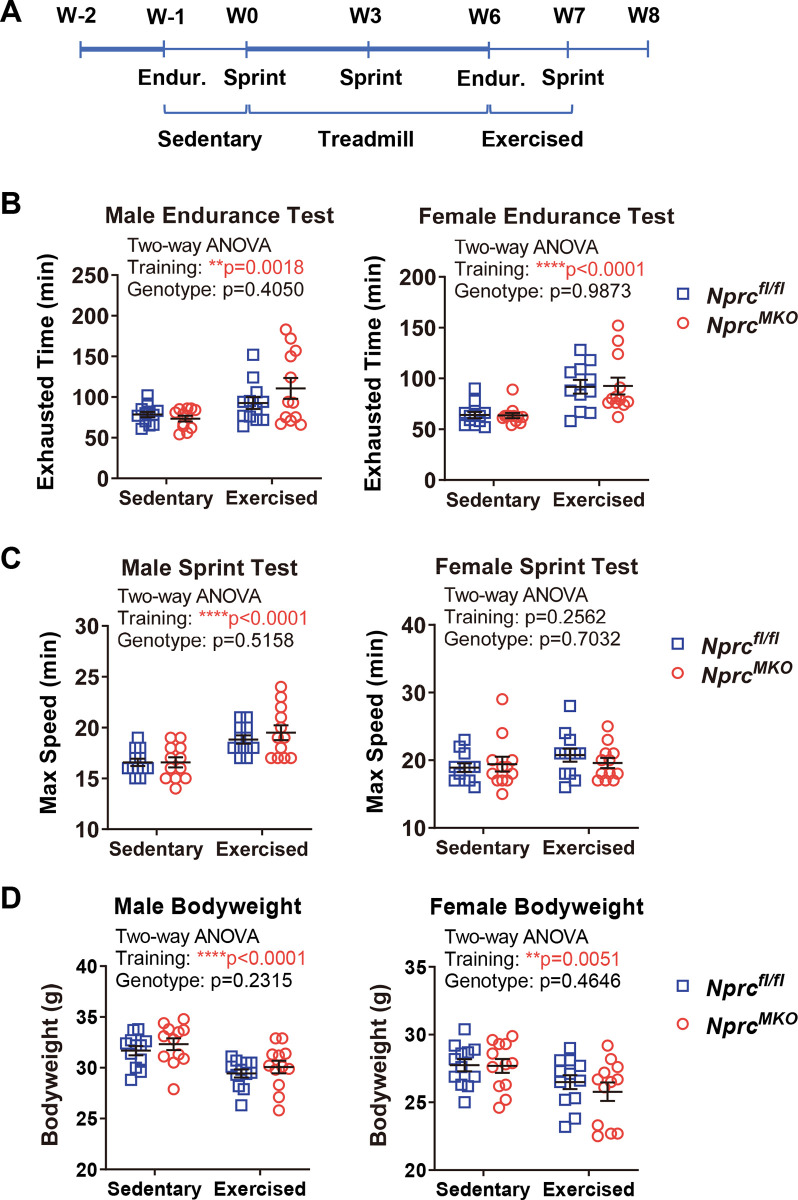
Exercise performance after 6 weeks of treadmill training. (A) Timelines of treadmill training, endurance (Endur.) and sprint exercise tests. Ten-month-old *Nprc* MKO and control male mice were trained on a treadmill at a speed of 60% of their maximal running speed for six weeks. (B, C) Mice were subjected to (B) sprint exercise test and (C) endurance exercise test at baseline (Sedentary) and after treadmill training (Exercised) to determine their maximal running speed and exhausted running time, respectively. (D) The body weight of each group of mice at baseline and after treadmill training. Two-way ANOVA, ** p < 0.01; **** p < 0.0001; and p>0.05, no statistical significance.

**Fig 6:**
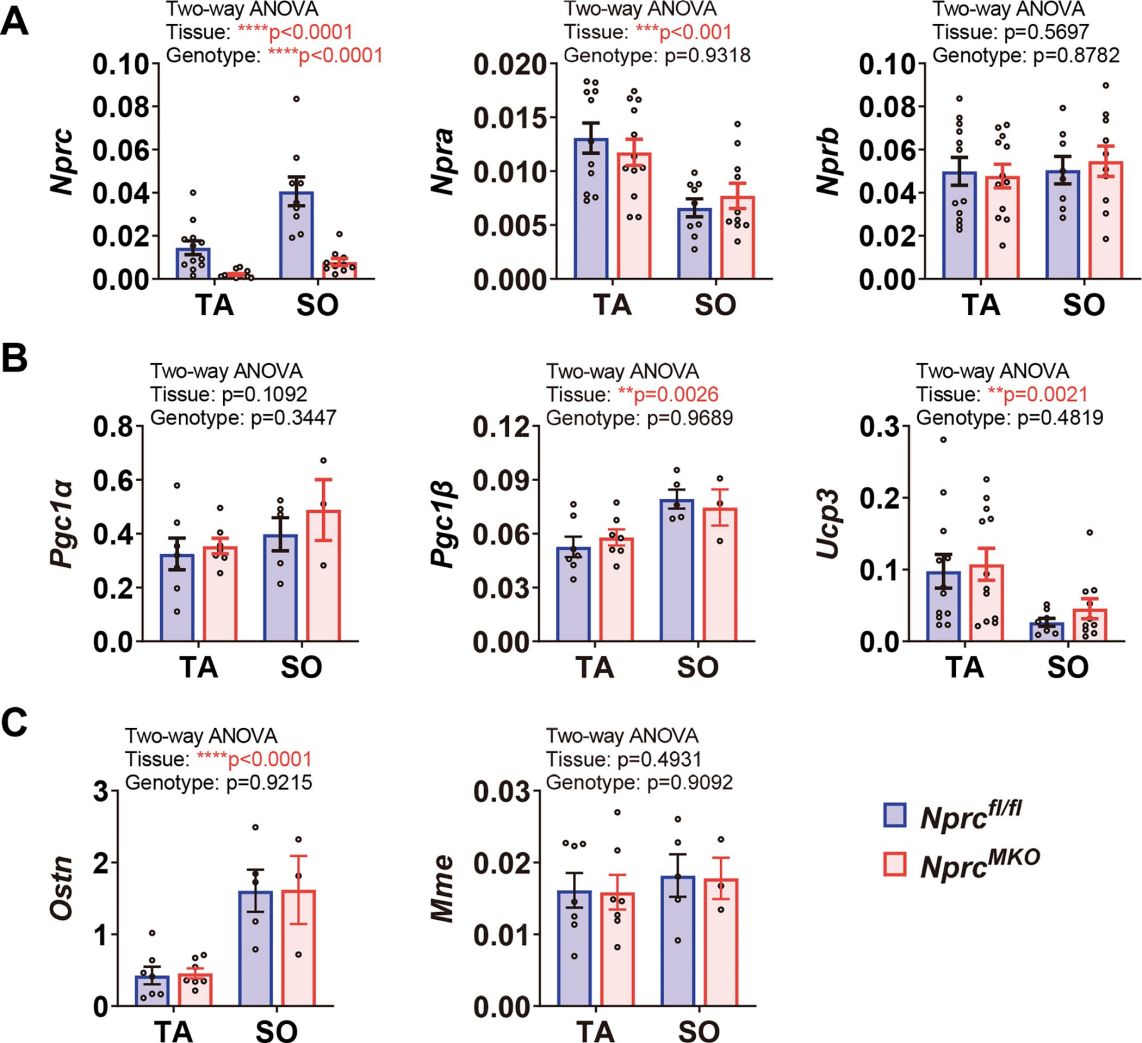
Gene expression profile in skeletal muscle of mice after treadmill training. The mRNA levels of (A) NP receptor genes *Nprc*, *Npra* and *Nprb*, (B) *Pgc1α*, *Pgc1β*, and *Ucp3*; and (C) osteocrin (*Ostn*) and neprilysin (*Mme*) in the tibialis anterior (TA) and solus (SO) of control and *Nprc* MKO mice. Two-way ANOVA, ** p < 0.01; *** p < 0.001; **** p < 0.0001; and p>0.05, no statistical significance.

Previous studies have shown that exercise performance declines during the human life span and in aged mice [23, 24]. Since we did not observe a robust improvement in the exercise capacity between *Nprc* MKO and control mice in relatively young cohorts, we next asked whether *Nprc* MKO mice would have better exercise capacity when older. A cohort of 1-year-old male and female mice were trained on treadmills for six weeks (**Fig 5A**). In the males, six-week treadmill training greatly reduced their bodyweight and improved the exercise performance of both *Nprc* MKO and control mice, with increased maximal running speed in the stress test and longer running distance in the endurance test, compared to the baseline (**Fig 5B–5D**). In the females, six-week treadmill training also results in body weight loss and increased running distance in the endurance test, but the improvement in maximal running speed in the stress test was not as robust as that of the male mice (**Fig 5B–5D**). However, compared to the control mice, we did not observe any greater improvement in exercise performance in *Nprc* MKO mice (**Fig 5B–5D**). In addition, as shown in **Fig 6** the gene expression profile was also comparable between *Nprc* MKO and control mice as well, including NP and NP receptors, *Pgc1α*, *Pgc1β*, *Ucp3*, and Osteocrin (*Ostn*). *Ostn*, also known as musclin [25], is able to competitively bind to *NPRC* to act as an inhibitor of NP clearance [26], and thus increase cGMP signaling through NPRA and NPRB. Proteolysis of NPs by neprilysin (NEP) typically acts in tandem with NPRC as an extracellular NP degradation pathway [2]. In both cases their expression was not different between genotypes.

## Discussion

Evidence from human and rodent studies suggests that natriuretic peptide signaling has an important role in skeletal muscle metabolism. For example, chronic BNP infusion has been shown to improve glucose homeostasis, insulin sensitivity, and increase lipid oxidative capacity in the skeletal muscle tissue of obese and Type 2 diabetic mice [27]. In addition, mice with global deletion of the *Nprc* gene (*Npr3*) have increased skeletal muscle fatty acid oxidation [22]. In humans, NPs have been reported to play a role in the promotion of mitochondrial biogenesis and oxidative respiration in skeletal muscle [9, 20]. The NPRC receptor, along with the enzyme neprilysin, is responsible for the vast majority of clearance and degradation of circulating NPs [2]. We previously showed that mice lacking *Nprc* in their adipose tissue had improved NP signaling and relative protection from diet-induced obesity [28]. Based on these combined observations we hypothesized that *Nprc* deletion in skeletal muscle would enhance NP signaling for metabolic benefits and result in improved exercise performance. However, while exercise training in the mice significantly increased their exercise performance and endurance, our data did not show a greater improvement in *Nprc* MKO relative to floxed control mice regarding their endurance or sprint running capacity either under sedentary conditions or after exercise training, in both young and aged mice. This was a bit unexpected given the effects observed in muscle of *Nprc-/-* mice [22], and suggests that NPRC deletion in skeletal muscle might not exert a major physiological consequence, at least for exercise capacity. The comparable exercise performance between *Nprc* MKO and floxed control mice raises the possibility that a compensatory mechanism may exist to counteract the loss of NPRC, serving to maintain the homeostasis of NP signaling in skeletal muscles. In addition, other metabolic perturbations such as high-fat diet feeding, while not differentially affecting body weight, composition or glucose tolerance between genotypes [7], may nevertheless reveal a difference in exercise capacity, which we did not study. Endogenous *Nprb* mRNA expression levels increase substantially in differentiated C2C12 myotubes (**Fig 1**), suggesting a role for the CNP-NPRB pathway in regulating myocyte metabolism and function. In other studies [28], localized measurements of the gradient between arterial and veinous CNP concentrations across the femoral vein in humans found that musculoskeletal tissue made a small but measurable contribution to plasma CNP levels in the body. Unlike ANP and BNP, CNP is not a cardiac hormone and is present at extremely low levels in circulation, portraying CNP-NPRB signaling as a relatively localized pathway for NP action in skeletal muscle [29]. Further study with loss of function mouse models, such as *Npra* or *Nprb* skeletal muscle knockout mice, will be necessary to clarify the contribution of different NP signaling pathways to skeletal muscle metabolism and exercise physiology in mice.

## Supporting information

S1 Fig Uncut Western blot images related to Fig 1D. Uncut Western blots of p-VASP, VASP and β-actin related to Fig 1D. Red rectangles indicate the sections of image in the Figure.
(TIF)

S2 Fig Uncut Western blot images related to Fig 1E. Uncut Western blots of p-VASP, VASP and β-actin related to Fig 1E. Red rectangles indicate the sections of image in the Figure.
(TIF)

S1 Table Sequences of QPCR primers. (DOCX)
