# Supplementary figures and images for: Exercise performance is not improved in mice with skeletal muscle deletion of natriuretic peptide clearance receptor
Source: PLoS One. 2023 Nov 2;18(11):e0293636. doi: 10.1371/journal.pone.0293636 (PMC10621814; doi:10.1371/journal.pone.0293636)

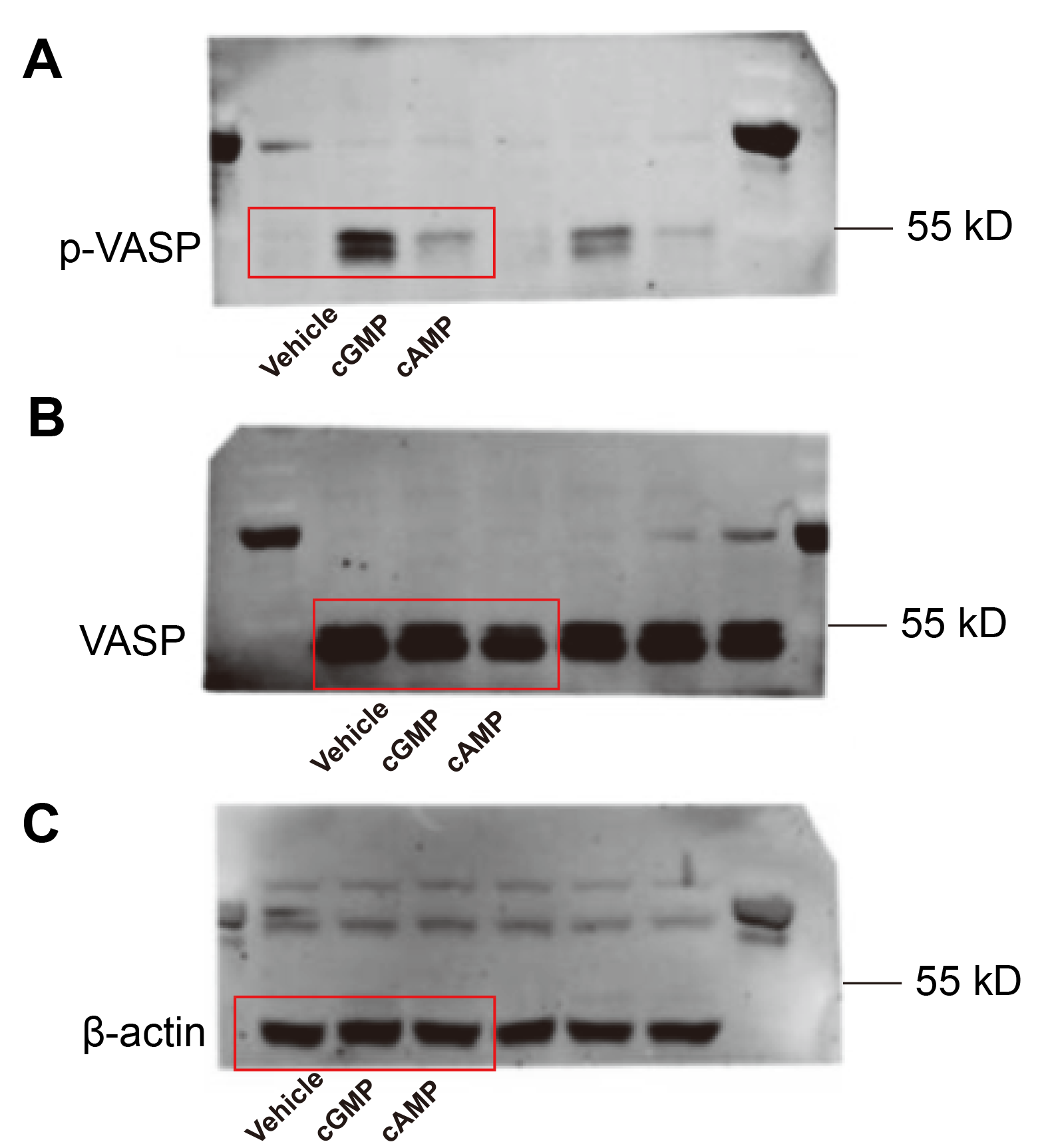

Supplement: S1 Fig — Uncut Western blots of p-VASP, VASP and β-actin related to Fig 1D. Red rectangles indicate the sections of image in the Figure. (TIF) [file pone.0293636.s001.tif]

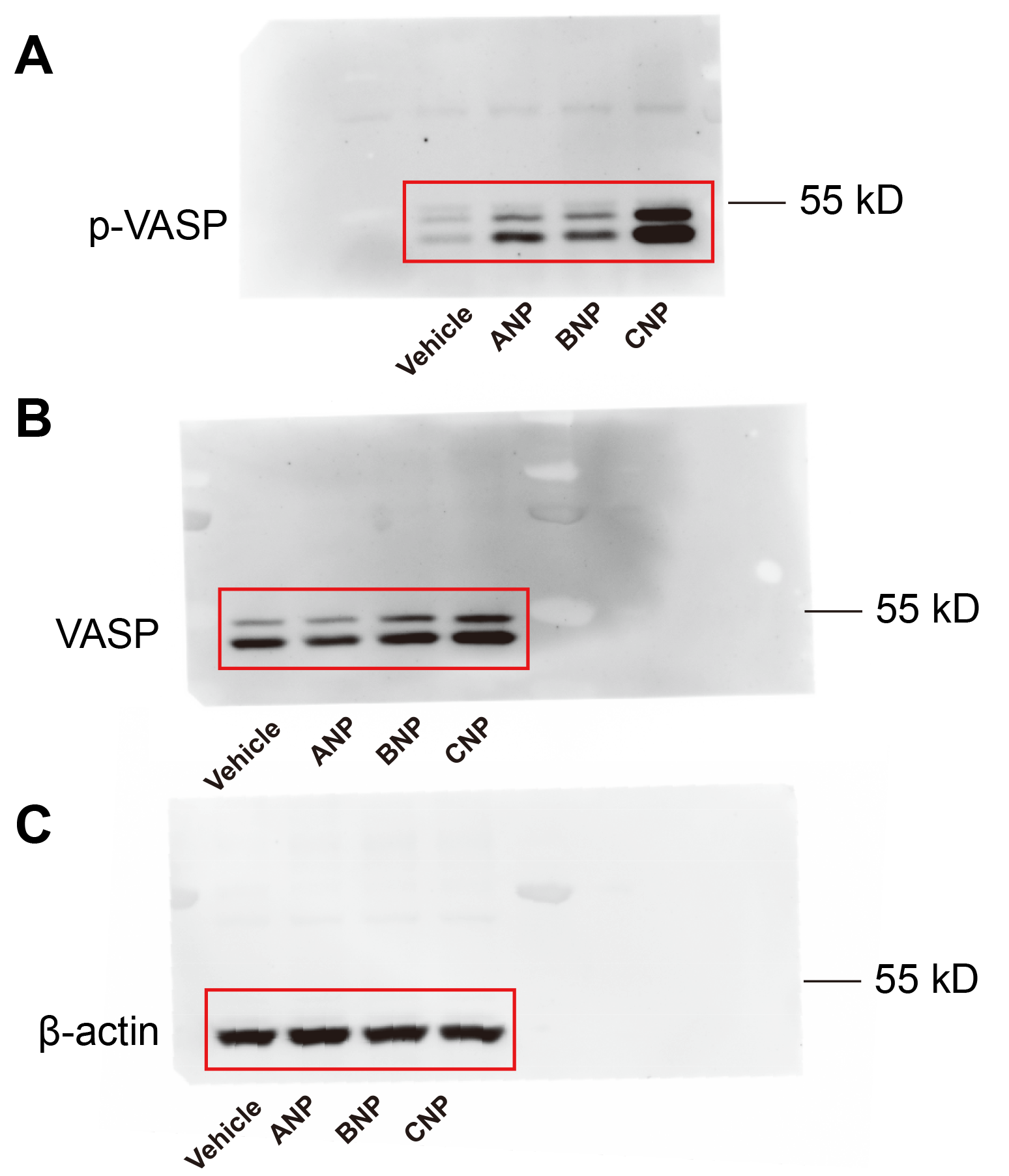

Supplement: S2 Fig — Uncut Western blots of p-VASP, VASP and β-actin related to Fig 1E. Red rectangles indicate the sections of image in the Figure. (TIF) [file pone.0293636.s002.tif]
