# Supplementary material for: Exercise performance is not improved in mice with skeletal muscle deletion of natriuretic peptide clearance receptor
Source: PLoS One. 2023 Nov 2;18(11):e0293636. doi: 10.1371/journal.pone.0293636 (PMC10621814; doi:10.1371/journal.pone.0293636)
Supplement: S1 Table — (DOCX) [file pone.0293636.s003.docx]

**Supplementary Data**

**Table S1. Sequences of QPCR primers.**

| **Genes** | **Fwd (5’-3’)** | **Rev (5’-3’)** |
| --- | --- | --- |
| *Npra* | TGGAGACACAGTCAACACAGC | CGAAGACAAGTGGATCCTGAG |
| *Nprb* | GAAGGCCTGGACCTCAGTC | TCAGTTGTGTCCGGTCAATG |
| *Nprc* | AGCTGGCTACAGCAAGAAGG | CGGCGATACCTTCAAATGTC |
| *Myod* | agcactacagtggcgactca | ggccgctgtaatccatcat |
| *Myog* | ccttgctcagctccctca | tgggagttgcattcactgg |
| *Pgc1α* | CGGAAATCATATCCAACCAG | TGAGAACCGCTAGCAAGTTTG |
| *Pgc1β* | ctccagttccggctcctc | ccctctgctctcacgtctg |
| *Ucp1* | GGCCTCTACGACTCAGTCCA | TAAGCCGGCTGAGATCTTGT |
| *Neprilysin* | GCCAAAGCAAGCAGCTAAAG | CTGATTTCGGCCTGAGGAATAA |
| *Osteocrin* | ccatggatcggattggtaga | tctgtgccatctcacacaagt |
